# Trade-Off Analysis for Array Configurations of Chipless RFID Sensor Tag Designs

**DOI:** 10.3390/s25061653

**Published:** 2025-03-07

**Authors:** Likitha Lasantha, Biplob Ray, Nemai Karmakar

**Affiliations:** 1 Department of Electrical and Computer Systems Engineering, Monash University, 14 Alliance Lane, Clayton, VIC 3800, Australia; nemai.karmakar@monash.edu; 2 School of Engineering and Technology, Central Queensland University, 120 Spencer St, Melbourne, VIC 3000, Australia; b.ray@cqu.edu.au

**Keywords:** array configurations, chipless radio frequency identification (RFID), detectability, detection performance, radar cross section (RCS)

## Abstract

The accurate detection and reliable performance of chipless radio frequency identification (RFID) tags and sensors present significant challenges due to their inherently low radar cross section (RCS) and pronounced mutual coupling effects. These limitations adversely influence the quality (Q) factor and overall detectability, complicating the optimisation of chipless RFID systems for practical applications. This study investigates the performance characteristics and trade-offs among RCS, Q-factor, and detectability in Pi-shaped array configurations of chipless RFID tags. A comprehensive analysis of various array configurations is conducted, supplemented by a link budget evaluation to elucidate how different array structures impact system performance. The simulation results reveal that planar arrays outperform linear arrays in both RCS and Q-factor, highlighting essential trade-offs between tag identification range and angular coverage, which are influenced by array size and electromagnetic coupling. The findings emphasise optimising resonance quality and scattering efficiency to tailor chipless RFID systems for specific application requirements. This research provides valuable insights into the design and operation of chipless RFID arrays, contributing to their advancement in practical applications.

## 1. Introduction

Radio frequency identification (RFID) technology has gained significant interest in the past few decades due to its potential applications in various fields [1]. These traditional RFID tags use a microchip and a battery to store information and connect to wireless networks [2]. However, these wireless tags can be expensive, difficult to incorporate with wearable devices, and difficult to operate in harsh environments. As a result, chipless RFID technology has emerged as a promising alternative, as it can be produced at a lower cost and with greater flexibility in various applications, such as supply chain management [3], healthcare [4], environmental monitoring [5], food packaging [6], and structural health monitoring [7].

Unlike traditional RFID tags, chipless RFIDs do not have a microchip or energy source. Instead, they use unique patterns of resonant structures to encode sensing and identification (ID) information and extract energy from the interrogation signal. These patterns can be detected using a reader that transmits an electromagnetic (EM) signal to the tag, causing it to resonate and retransmit a unique EM signature that can be decoded to extract information [8]. Therefore, the resonator structure, the size of the tag, the quality (Q) factor, and the radar cross section (RCS) are the critical parameters that determine the performance and precision of the chipless RFID system [9].

In chipless RFID systems, the RCS parameter crucially influences the magnitude of the reflected signal, thus affecting the overall system performance [10]. In other words, it determines the magnitude of the reflected signal; therefore, the detectability of the chipless tags depends on it. In general, the RCS of chipless RFID tags depends mainly on the size of the tag [11]. Therefore, designing an optimised tag with a high RCS is essential to achieve reliable and accurate performance in real-world applications. Array configurations are the most promising approach to improving the RCS. This paper aims to analyse linear and planar array configurations to improve the RCS of the chipless RFID tag.

In addition, the Q-factor also plays an important role in determining the resolution and detectability of chipless RFID tags and sensors [12]. A high Q-factor creates sharp spectral dips in chipless RFID sensors, enhancing sensing precision and resolution. However, a low Q-factor generates broad spectral dips, diminishing sensitivity and accuracy. Similarly, a high Q-factor improves readability and detectability in chipless RFID tags by producing separate spectral signatures. However, a low Q-factor may lead to broader and not-so-clear responses, thereby making identification uncertain. Thus, an optimal balance in the case of chipless RFID sensors and tags is demanded in order to achieve all-around improvement and consistency in all applications.

Therefore, this study will further investigate the Q-factor and linear and spherical detectability analysis with the array configurations. However, despite the widespread use of array configurations, the proper theoretical and experimental analysis still needs to be improved to understand the trade-offs between these parameters. Therefore, this paper addresses the identified gaps and proposes solutions. The novel contributions of this paper are as follows:

1. Experimental validation of the performance of chipless RFID tags using linear and planar array configurations.
2. Examination of detectability highlights trade-offs between tag identification range and angular coverage.
3. Comprehensive analysis elucidates trade-offs among parameters such as linear detectability, spherical detectability, RCS, and Q-factor.
4. Providing guidance for optimising the design and operation of chipless RFID tags based on planar array configurations.

The paper is organised as follows: In Section 2, the RCS is introduced, and techniques that can be used for its improvement are discussed. Section 3 presents a link budget analysis of chipless RFID arrays. Section 4 explains the design of resonators for unit-cell, linear, and planar arrays. Section 5 provides the analysis of a simulation study of the unit cell and array configurations, while in Section 6, the experimental validation and discussion are presented. In Section 7, the trade-off analysis is discussed. Finally, the paper is concluded in Section 8.

## 2. RCS of Chipless RFID Arrays

The concept of the backward link in a chipless RFID tag is based on signal scattering, similar to how a radar system operates [13]. Essentially, the RCS of a chipless RFID tag refers to the ratio of the reflected signal power compared to an ideally smooth sphere with a 1 m^2^ cross-sectional area. This value is usually represented as m^2^ or normalised value dBsm. In simpler terms, the RCS of a chipless RFID tag determines how effectively it can reflect signals to the reader. This is important for maintaining reliable communication between the tag and the reader.

**Figure 1:**
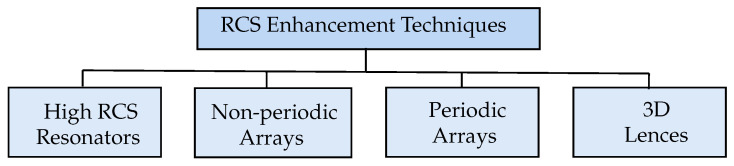
RCS enhancement techniques.

There are several ways to improve the RCS of chipless RFID tags, as shown in Figure 1, such as using resonators with a higher RCS value, but it shows a slight improvement as expected [14]. In addition, the same resonator can be placed in a non-periodic arrangement to increase the RCS. However, this arrangement is shown to not considerably increase the RCS level [15,16]. Moreover, 3D lenses can also be used to improve the RCS of the chipless tag, but that improvement is minimal and utilises more resources [17]. Therefore, the most promising way to increase the chipless tag’s RCS is to periodically replicate a unit-cell tag or resonator, either linearly or planar array configurations. Although these replications do not increase the tag data encoding capacity, they significantly enhance the detection probability by increasing the RCS level [18].

In [19], a configuration is presented, where three 13-bit slot ring resonators (SRRs) are organised into a linear 1 × 3 array with an inter-element distance of 1 mm, resulting in an enhanced RCS. This arrangement exhibits a 5 dBsm improvement in RCS compared to an individual SRR. Similarly, another work introduces a 3 × 3 planar array of crack sensors based on compass-shaped patch resonators. This array design increases the coverage area for crack monitoring and improves the RCS response of the sensor array by approximately 10 dBsm when compared to a single cell [20]. Additionally, in [21], a configuration involving a 4 × 4 planar array of 4-bit SRRs is proposed to extend the reading distance and amplify the RCS value by +20 dBsm in contrast to the RCS of a unit cell tag. So far, all the experiments have concentrated solely on improving RCS through array configurations. However, while RCS is enhanced, other critical parameters, such as the Q-factor and spherical detectability, are reduced. Therefore, balancing these parameters to achieve the optimal outcome is essential.

### 2.1. RCS Modelling

When a tag array is illuminated by a linearly polarised instantaneous E-field wave ($E_{i}$), a reflected wave ($E_{r}$) is observed at the receiver. The following mathematical formula gives the relationship between the RCS of the chipless tag, incident, and reflected waves [22]:

(1) $$\sigma_{\mathrm{tag}}\left(f,\psi \right)=\lim_{d\to \infty }4\pi d^{2}\frac{{\left|E_{r}\left(f,\psi \right)\right|}^{2}}{{\left|E_{i}\left(f,\psi \right)\right|}^{2}}$$

where $\sigma_{\mathrm{tag}}\left(f,\psi \right)$ is the RCS of the chipless tag, *f* is the frequency of the chipless tag, ψ is the transpose vector of the angles of incident and reflected waves, which equal to ${\left[\theta_{i},\phi_{i},\theta_{r},\phi_{r}\right]}^{T}$. $\theta_{i,r}$ is equal to the elevation, $\phi_{i,r}$ is equal to the azimuth of the incident and reflected waves, and *d* is the distance between the chipless tag and the reader.

**Figure 2:**
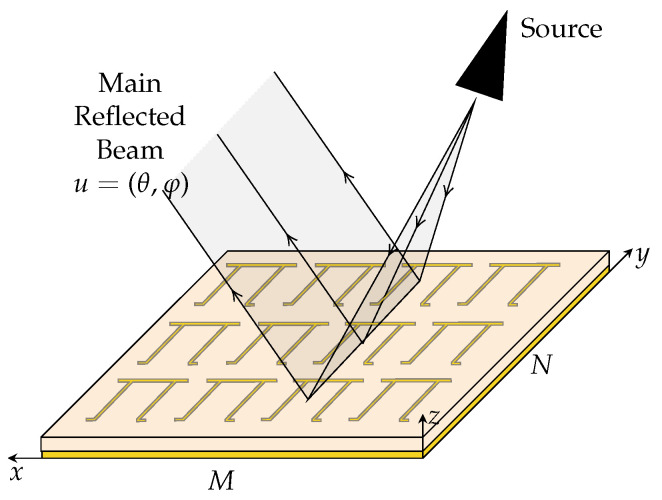
M×N Chipless RFID array in a rectangular coordinate system.

When considering the scattering intensity of a chipless array in a particular direction, it is directly proportional to the physical size of the scattering array. Additionally, it depends on the interference between the different scattering element contributions on the array. Since the chipless array combines various unit cells, each with its unique reflection coefficient, the overall scattering pattern at a given frequency can be calculated similarly to an antenna array. Therefore, the monostatic RCS of the tag array is calculated by weighting the normalised pattern towards the direction with the same RCS as a plate of equal area as the total scatterer [15]:

(2) $$\sigma_{\mathrm{array}}\left(f,\psi \right)=\frac{4\pi A^{2}}{\lambda^{2}}{\left(\frac{\left|E_{r}\left(f,\psi \right)\right|}{MN\cos(\theta_{r})}\right)}^{2}$$

where *A* is the total geometric area of the array, λ is the wavelength, and *M* and *N* are the elements of the array in the x and y directions as shown in Figure 2.

### 2.2. RCS Measurement Techniques

**Figure 3:**
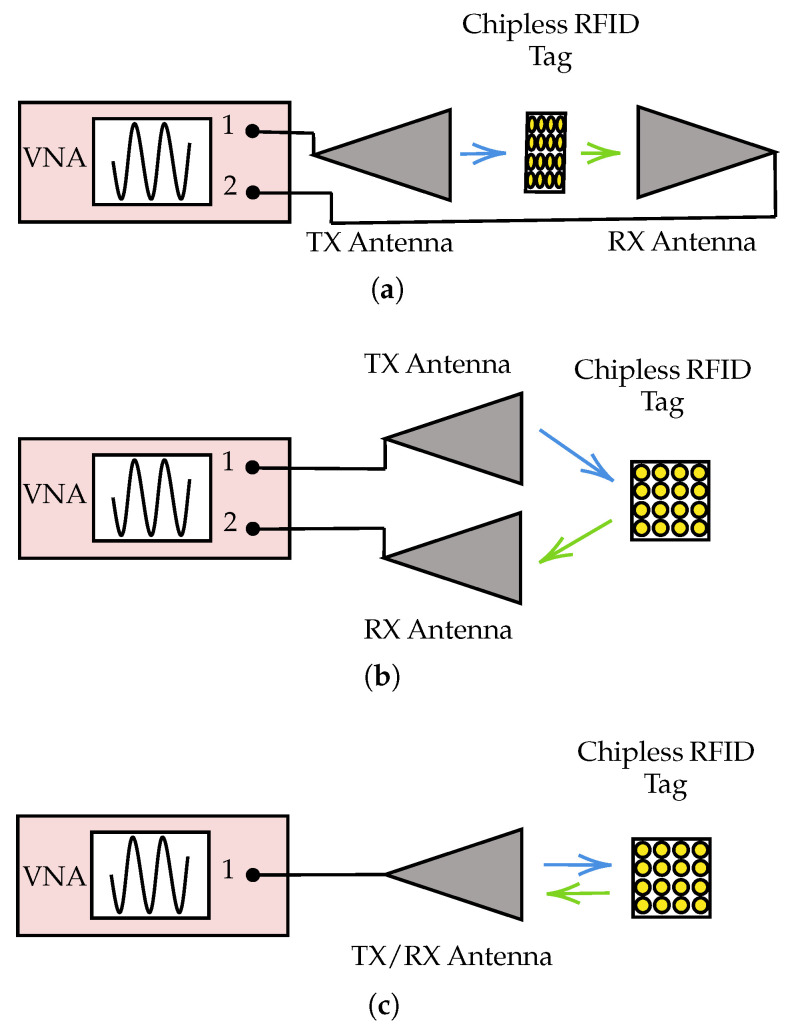
Common RCS experimental setups. (**a**) Bistatic configuration—Dislocated. (**b**) Bistatic configuration—Collocated. (**c**) Monostatic configuration.

A bistatic configuration, shown in Figure 3a,b, is commonly used to measure RCS. Calibration of the RFID sensing system is necessary to eliminate unwanted background noise. Equation (3) can be used to estimate the calibrated RCS value of a chipless RFID tag [23].

(3) $$\sigma_{\mathrm{tag}}={\left[\frac{S_{21}^{\mathrm{tag}}-S_{21}^{\mathrm{background}}}{S_{21}^{\mathrm{ref}}-S_{21}^{\mathrm{background}}}\right]}^{2}\;\sigma_{\mathrm{ref}}$$

where $S_{21}^{\mathrm{tag}}$ is the response of the tag, $S_{21}^{\mathrm{background}}$ is the response without the tag, and $S_{21}^{\mathrm{ref}}$ is the response of the known object, which can be calculated as explained in [11].

In the case of a monostatic antenna configuration, as shown in Figure 3c, there is one standard antenna to transmit and receive signals. Therefore, the term $S_{21}$ in the Expression (3) can be replaced with $S_{11}$.

## 3. Link Budget of Chipless RFID Arrays

The link budget analysis is a crucial parameter used to evaluate the effectiveness of a chipless RFID system. It determines the total gains and losses in the communication framework, which helps establish the maximum distance possible for reliable communication. Chipless RFID systems do not have an integrated circuit (IC), which makes the link budget analysis different from conventional RFID setups. In chipless RFID, wireless communication between a reader and a tag occurs through the backscatter of electromagnetic waves. However, this scenario introduces complex design challenges due to the intricate interplay among the reader, tag, and the radio channel.

The interactions between the reader, tag, and radio channel are complex and can affect the power received by the tag ($P_{in}$) and the power backscattered into the reader ($P_{bs}$). These interactions can limit the received or backscattered power ($P_{r}$) to levels below a critical threshold, which equals the sensitivity of the reader ($P_{s}$). If the power falls below this threshold, the reader may not detect the backscattered signal, and communication will become unreliable. Therefore, accurately predicting received powers is essential for the effective design and operation of modulated backscatter systems in chipless RFID. The link budget equation for chipless RFID can be expressed as

(4) $$\mathrm{Link}\;\mathrm{Budget}=P_{t}+G_{t}-{\mathrm{FSPL}}_{1,2}+\sigma_{\mathrm{tag}}-G_{r}-P_{s}$$

where $P_{t}$ is the transmitted power by the reader, $G_{t}$ is the gain of the RFID transmitting antenna, FSPL_1,2_ is the free-space path loss in the forward and backward link, $\sigma_{\mathrm{tag}}$ is the RCS of the chipless tag, $G_{r}$ is the gain of the RFID receiving antenna, and $P_{s}$ is the sensitivity of the reader.

A positive link budget indicates that the received power at the RFID reader is equal to or greater than the receiver’s sensitivity ($P_{s}$), ensuring successful detection and reliable communication with the chipless RFID tag. In contrast, a negative link budget means the received power is below the required sensitivity, leading to unreliable detection or complete communication failure.

### The Role of Chipless RFID Arrays in Link Budget

**Figure 4:**
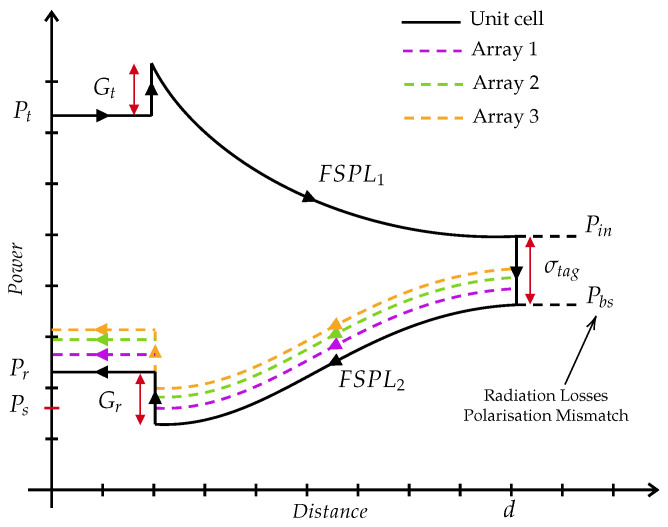
Link budget of a chipless RFID array system.

Figure 4 illustrates the link budget of a chipless RFID array system. The RCS of a chipless RFID tag ($\sigma_{\mathrm{tag}}$) plays a crucial role in link budget analysis, particularly in understanding how efficiently the tag scatters incident power back to the reader. In the unit cell chipless tag, the RCS is relatively small compared to the array configurations, which reduces the tag scattering effect. Then, it reduces the power received by the RFID reader. Moreover, when considering the chipless RFID array configurations, it is evident that RCS increases when the number of elements in the array increases (i.e., Array 1 to Array 3). Therefore, it also increases the power received by the reader $P_{r}$. However, the array enhanced from Array 1 to Array 3 shows no linear variation. This is because when the elements in the array increase, the polarisation mismatch and radiation losses also increase, leading to proportionally reduced received power to the reader as shown in Figure 4.

In the dislocated bistatic configuration, as shown in Figure 3a, the received power to the chipless RFID reader can be theoretically estimated using the following formula [24]:

(5) $$P_{r}=\frac{P_{t}G_{t}G_{r}\lambda^{4}X_{f}X_{b}\sigma \left(\theta ,\phi \right)}{{\left(4\pi \right)}^{4}r_{f}^{2}r_{b}^{2}B_{f}B_{b}F}$$

where λ is the wavelength of the resonance frequency, $X_{f,b}$ is the forward and reverse-link polarisation mismatch, $r_{a,b}$ is the tag-to-reader and reader-to-tag distances (m), $B_{f,b}$ is the forward and reverse blockage losses, and *F* is the bistatic fade margin.

In the collocated bistatic configuration, as shown in Figure 3b, $X_{f}=X_{b}$, $r_{f}=r_{b}$, and $B_{f}=B_{b}$. In addition to these parameters, $G_{t}=G_{r}$ happens in a monostatic configuration since it uses only one antenna as shown in Figure 3c.

## 4. Chipless RFID Array Design

Here, a novel Pi-shaped chipless RFID tag design is presented. It is then used to configure the linear and planar array tags.

### 4.1. Unit Cell Tag

**Figure 5:**
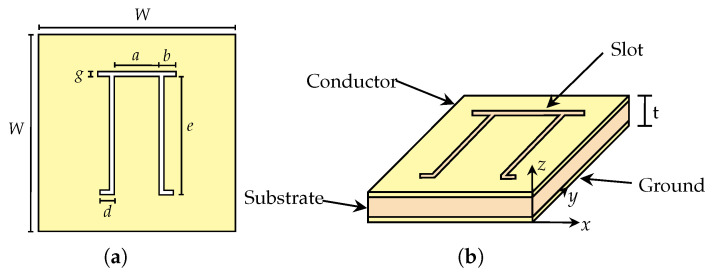
A novel Pi-shaped unit cell chipless RFID tag (*a* = 3.6 mm, *b* = 1.4 mm, *g* = 0.5 mm, *d* = 1.2 mm, *e* = 12 mm, *W* = 20 mm and *t* = 0.5 mm). (**a**) Plan view. (**b**) Perspective view.

A novel chipless RFID tag design features a resonator shaped like the mathematical symbol Π, as illustrated in Figure 5a, with all dimensions provided in Figure 5b. The reason for choosing an open resonator with a ground plane is that it exhibits a higher Q-factor than a closed-shaped resonator as summarised in Table 1. In Table 1, we compare the Q-factors of the most common chipless RFID resonators in the literature by simulating them on the same Taconic TLX-8 substrate with dimensions of 20 × 20 mm^2^ and under the same simulation environment. The Q-factor is calculated using the following equation [25]:

(6) $$Q\;\mathrm{Factor}=\frac{f_{0}}{BW_{-3\mathrm{dB}}}$$

where $f_{0}$ is the frequency at which the spectral dip occurs (i.e., resonance frequency), and $BW_{-3\mathrm{dB}}$ is the −3 dB impedance bandwidth at $f_{0}$.

This definition is commonly used for transmittance measurements, where the resonance dip indicates energy absorption; however, in the case of backscattering (RCS) responses, multiple reflections and anti-phase interactions significantly affect the bandwidth. Reflected signal components can interfere destructively, leading to deep spectral nulls where the RCS nearly drops to zero, resulting in highly narrow bandwidths and causing the Q-factor to tend toward infinity. The interactions among metal structures, resonators, and array configurations influence the spectral variations. Measuring the bandwidth from the RCS response rather than from transmittance is crucial to accurately defining the Q-factor for backscattering scenarios. Therefore, we use the backscattering Q-factor for this study.

**Figure 6:**
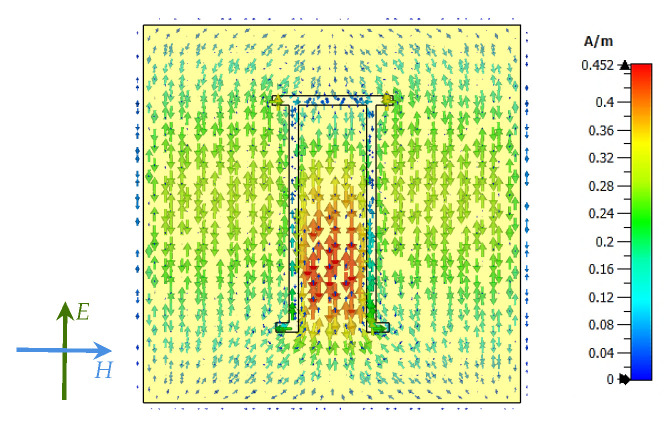
Surface current distribution of Pi-shaped resonator at resonance frequency, 4.78 GHz.

The design of the Pi-shaped resonator is inspired by the U-shaped resonator, which exhibits a higher Q-factor, a smaller footprint, and the ability to resonate in a ground plane [25]. This tag is initially designed to operate at a frequency of 4.8 GHz, which belongs to the ultra-wideband (UWB) frequency range. However, it is further refined to achieve a better Q-factor, which results in adjusting the resonance frequency to 4.78 GHz. In our design, we modify the U-shaped resonator by inverting it to form a Pi-shaped one and stretching both the top and bottom resonator tips. This modification allows for better surface current distribution across the resonator surface as shown in Figure 6.

**Figure 7:**
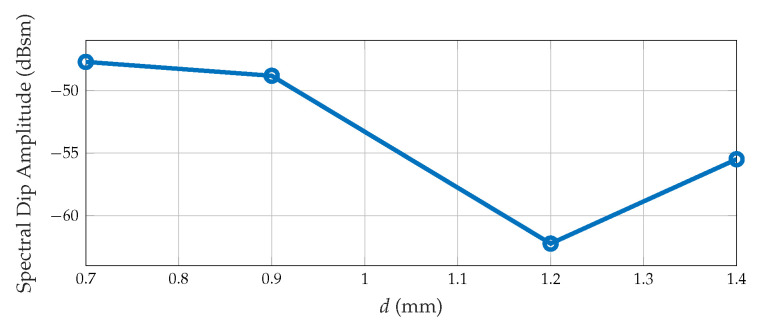
The spectral dip’s amplitude level variation of the Pi-shaped resonator arm length *d* changes from 0.7 to 1.4 mm when *b* is equal to 1.4 mm.

Furthermore, the upper and lower tips in our Pi-shaped resonator design have different lengths, denoted by *b* and *d*, respectively (e.g., *b* ≠ *d*). This asymmetrical configuration enhances the spectral dip’s amplitude, improving the detectability of the resonator in the frequency signature as shown in Figure 7. The level of the spectral dip’s amplitude increases when the length of the arm *d* increases from 0.7 to 1.4 mm. When the resonator arm length *d* equals *b*, the spectral dip’s amplitude level decreases. Therefore, this asymmetrical configuration benefits resonator detectability using the frequency signature.

Similarly, the thickness of the Taconic TLX-8 substrate, with a dielectric constant of 2.55 and a loss tangent of 0.0019, is optimised to 0.50 mm to enhance the resonator Q-factor further. Moreover, increasing the Q-factor reduces the variation in resonance frequency over a significant distance, allowing for a more accurate analysis of the RCS variation in the array configurations [26]. A higher Q-factor also improves the overall performance of the chipless RFID tag, as it minimises energy loss [25]. The resonance frequency of the novel resonator is derived using a non-linear regression using the model equation in [27], and it can be calculated using the following:

(7) $$f_{0}=\frac{c}{1.25L}\left(\sqrt{\frac{2}{\epsilon_{\mathrm{eff}}+1}}\right)\;\;\;\;\;\;\;\;\;\;[\mathrm{Hz}]$$

where *c* is the speed of light in vacuum, $\epsilon_{\mathrm{eff}}$ is the effective dielectric constant, and the effective length of the Pi-shaped resonator *L*,

(8) L=[a+2(b+d)]+2e          [m]

where *a*, *b*, *d*, and *e* are the dimensions of the resonator. For a better Q-factor, keep *a*, *b*, and *d* constant and only vary the dimension *e* to adjust the resonance frequency $f_{0}$ of the resonator.

**Table 1:** Summary of Q-factor comparison of common chipless RFID resonators.

Ref.	Resonator Type	Resonance Frequency (GHz)	Simulated Q-Factor
without Ground Plane			
[25]	U-shaped Slot Resonator	4.372	329
[28]	Asymmetric Circular Split Ring Resonator	5.760	182
[29]	Slot Ring Resonator	4.352	125
[30]	Cobweb Resonator	4.504	111
[31]	ELC Resonator	9.249	178
with Ground Plane			
[23]	Square Resonator	4.597	159
[32]	Tip Loaded Dipole Resonator	5.461	229
[33]	Triangular Patch Resonator	5.308	128
This work	Pi-shaped Resonator	4.780	4515

### 4.2. Linear Array Tags

**Figure 8:**
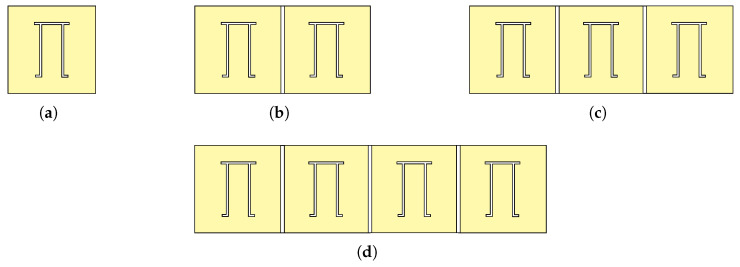
Linear array configurations of Pi-shaped resonator. (**a**) Unit cell. (**b**) 1×2 array. (**c**) 1×3 array. (**d**) 1×4 array.

The linear array configuration is a fundamental arrangement of unit cells in a straight-line formation characterised by the equal spacing between the unit cells. In this study, we use CST Microwave Studio to design unit cells and three specific linear configurations, namely, 1×2, 1×3 and 1×4 arrays as shown in Figure 8.

**Figure 9:**
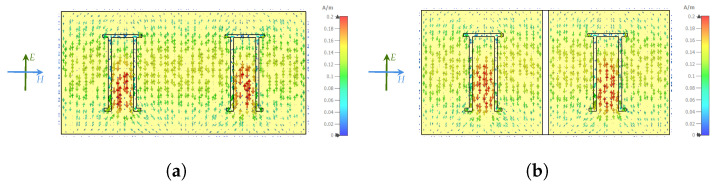
Surface current distribution of 2×2 array configuration. (**a**) Without removing copper strips. (**b**) Removing copper strips.

In addition, to mitigate the mutual coupling effect among the resonators in the array design, a copper strip with a width of 1 mm is intentionally removed between each resonator element. This deliberate removal of the copper strip concentrates the surface current distribution around the resonators and the ground plane, reducing mutual coupling and improving the array’s performance based on reflector array theory. According to Figure 9a, the surface current distribution is spread over all resonators, making the strong mutual coupling effect. However, in Figure 9b, the surface current distribution is controlled by removing these copper strips and mitigating the mutual coupling effect.

### 4.3. Planar Array Tags

**Figure 10:**
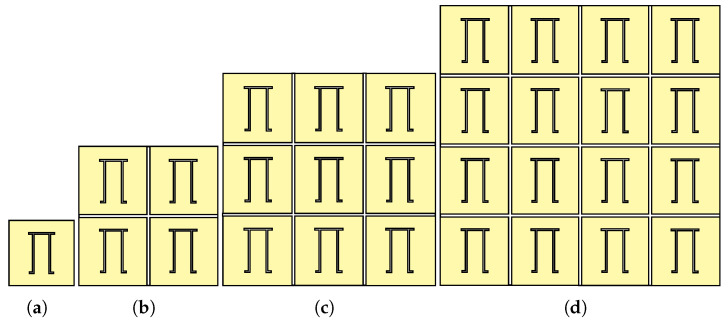
Planar array configurations of the Pi-shaped resonator. (**a**) Unit cell. (**b**) 2×2 array. (**c**) 3×3 array. (**d**) 4×4 array.

A planar array configuration is the arrangement of unit cells in a two-dimensional (2D) plane, typically in rows and columns. In this study, a unit cell and three planar array configurations (2×2, 3×3, and 4×4 arrays) are designed using CST Microwave Studio for the comparison of RCS as shown in Figure 10. The simulations provide information on the performance characteristics of each array, such as the radiation pattern, resonance frequency, and RCS. The array configurations are carefully designed and simulated in a delicate mesh size environment to obtain accurate RCS data, and the results are analysed in subsequent sections for further analysis.

## 5. Simulation Study of Array Configurations

In the CST Microwave Studio simulation configuration, the chipless RFID reader comprises a plane wave excitation and an RCS probe. These components are located at a distance of 30 cm from the tag. The plane-wave excitation transmits an interrogating signal that is linearly polarised and orientated in the z direction. Precisely, the electric field plane (E-plane) of the wave aligns with the y-axis, whereas the magnetic field plane (H-plane) aligns with the x-axis. The simulation covers a frequency span from 3.5 to 6 GHz and adopts an open boundary configuration in all spatial dimensions.

### 5.1. RCS Analysis

Upon activation by the plane wave, the RCS showcases a distinct behaviour tied to frequency. This behaviour is characterised by a notable dip or notch at a particular frequency. The origin of this notch can be attributed to the resonance of the Pi-shaped resonant structure present within the tag. At the specific resonance frequency, the Pi-shaped structure interacts with the excitation signal, leading to substantial energy absorption at these frequencies before any reflection occurs. This resonance phenomenon generates the frequency-selective response observed in the RCS.

**Figure 11:**
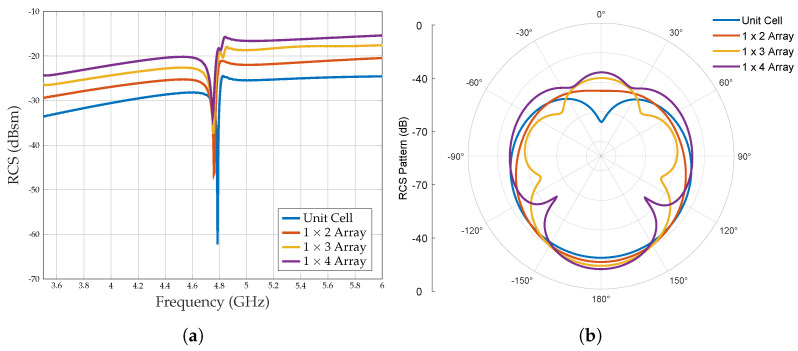
Simulated RCS response of the different linear array configurations. (**a**) Frequency response. (**b**) Scattered RCS pattern when ϕ = 90° at the resonance frequency.

When the designed unit cell tag is impinged by a plane-wave excitation signal, it shows a unique surface current distribution across the surface of the tag. The low current density is concentrated inside the Pi-shaped resonant structure, corresponding to the presence of capacitive properties. On the other hand, the highest distribution of surface current is shown between the two arms of the resonator structure, indicating the presence of inductive effects. The presence of inductive and capacitive characteristics distributed throughout a single resonant structure causes a significant resonance dip. It can be easily observed in the RCS frequency response of the unit cell tag as illustrated in Figure 11a.

The simulated RCS for various configurations of the linear array is presented in Figure 11a. The RCS of the unit cell tag is approximately −25 dBsm, which improves by +10 dBsm when configured as a 1×4 array. This trend indicates that the RCS level tends to increase with the number of elements in the array. A similar enhancement in RCS can be observed in the scattered RCS patterns shown in Figure 11b. Moreover, the resonance frequency demonstrates variations compared to the unit cell tag, with a noticeable shift towards lower frequencies (from 4.78 GHz to 4.75 GHz) as the number of array elements increases as detailed in Table 2.

The minor variations in resonance frequency between unit cell and array configurations arise from electromagnetic coupling among adjacent elements, which alters the effective inductance and capacitance. The expanded reflective surface area in arrays enhances the RCS and modifies resonance conditions, while structural mode reflections also contribute to frequency shifts. Additionally, variations in substrate properties, fabrication imperfections, and environmental factors, such as measurement alignment and external noise, can influence the frequency response. For larger arrays, increased radiation losses and a reduced Q-factor can affect the resonance stability, leading to slight frequency deviations compared to a single unit cell.

Finally, it is important to recognise that linear array configurations are sensitive to the reader’s location due to their lack of symmetry along the z-axis of the reader. Consequently, these linear array configurations may not be ideal for chipless RFID applications because of the resulting deviations in measurement parameters.

**Figure 12:**
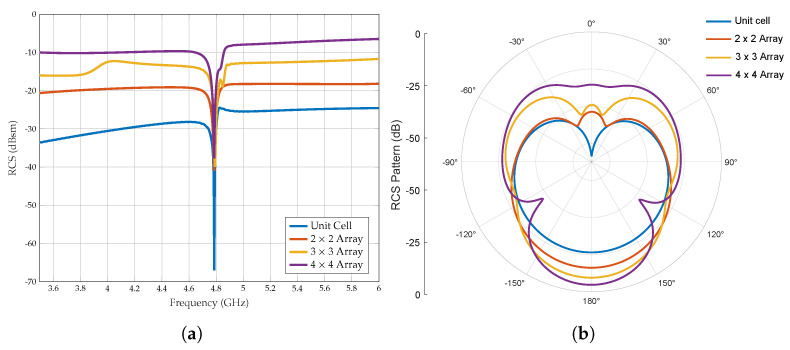
Simulated RCS response of the different planar array configurations. (**a**) Frequency response. (**b**) Scattered RCS pattern when ϕ = 90° at the resonance frequency.

A simulated RCS of the different configurations of planar arrays is shown in Figure 12a, and it is clear that the RCS increases as the number of cells in the array increases. Furthermore, planar arrays are symmetric in both the x and y directions; therefore, they are resilient to the reader’s small movements and show a constant resonance frequency. Moreover, as shown in Figure 12b, the scattered RCS patterns also show an improvement in the RCS level.

Additionally, as summarised in Table 3, the RCS value of the 4×4 array tag is almost 18 dBsm higher than that of the unit cell tag, demonstrating a significant enhancement in backscattering strength. Since the 1×4 linear array and the 2×2 planar array have the same footprint area, their total RCS values might be expected to be similar. However, the mutual coupling between antenna elements in the planar array influences the overall RCS, leading to differences in scattering characteristics. In the 2×2 configuration, each element interacts more strongly with its three adjacent elements, which alters the overall response of the array.

When the number of unit cells normalises the RCS, the efficiency of each configuration becomes clearer. The 1×4 array exhibits a normalised RCS of −9.02 dBsm/cell, whereas the 2×2 planar array has a lower normalised RCS of −24.02 dBsm/cell. This indicates that each unit cell in the linear array contributes more efficiently to backscattering than the planar array. The reduced efficiency in the planar array is attributed to stronger mutual coupling, where each element’s interaction with multiple neighbours impacts its scattering behaviour more significantly than in the linear arrangement.

#### Structural and Antenna Mode RCS of Array Configurations

**Figure 13:**
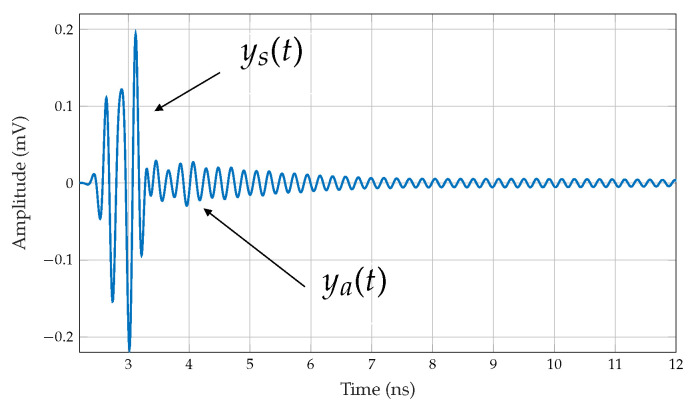
Backscattered structural, $y_{s}\left(t\right)$, and antenna, $y_{a}\left(t\right)$, mode signal of the chipless tag in the time domain.

In the context of a chipless RFID system, the signal received by the reader from a chipless tag, denoted as y(t), is expressed as the sum of three components in the time domain:

(9) $$y\left(t\right)=y_{r}\left(t\right)+y_{s}\left(t\right)+y_{a}\left(t\right)$$

where the transmitted pulse rejection $y_{r}\left(t\right)$ is the highest and first signal to arrive at the reader. The second component is the structural mode RCS, $y_{s}\left(t\right)$, related to the tag’s reflective surface. The antenna mode RCS component, $y_{a}\left(t\right)$, arrives last with lower strength and contains all data bit information about the chipless RFID tag [11]. Therefore, the structural and antenna mode RCS components are crucial in analysing the RCS of chipless tags, and the transmitted pulse rejection $y_{r}\left(t\right)$ is removed from the time domain signal for further analysis by employing a raised cosine window as depicted in Figure 13.

As the number of elements in an array configuration increases, the received signal strength of the structural mode RCS ($y_{s}\left(t\right)$) becomes more substantial due to the increased total reflective area. At the same time, the increased total reflective surface causes the tag structure to resonate itself, resulting in an additional spectral dip in the frequency signature. A time-domain analysis is conducted using a unit cell and a 4 × 4 array configuration to examine this phenomenon.

**Figure 14:**
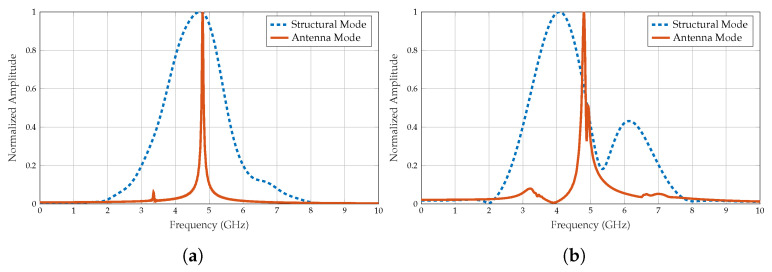
Normalised structural and antenna mode backscattered signal obtained using FFT algorithm. (**a**) Unit cell. (**b**) 4 × 4 array.

In the unit cell scenario, as shown in Figure 14a, the structural mode RCS component forms a Gaussian spectrum, indicating a uniform distribution of reflected energy across frequencies. Conversely, the antenna mode RCS component exhibits a pronounced peak, emphasising the resonant behaviour of the tag resonator at a specific frequency. This resonance is a crucial feature harnessed for encoding information.

Transitioning to the 4 × 4 array configuration, as shown in Figure 14b, the structural mode RCS displays deviations from the Gaussian spectrum, revealing the emergence of spectral dips, particularly around 5.20 GHz. This phenomenon results from the collective resonance and interference effects induced by the increased number of reflective elements in the array. The spectral dips in the structural mode RCS signal can be misinterpreted as a data bit. On the other hand, the antenna mode RCS component shows a prominent peak, highlighting the resonant behaviour of the tag at a designed frequency. Therefore, when designing chipless RFID arrays, it is necessary to consider the trade-off between the structural and antenna mode RCS.

### 5.2. Q-Factor Analysis

**Figure 15:**
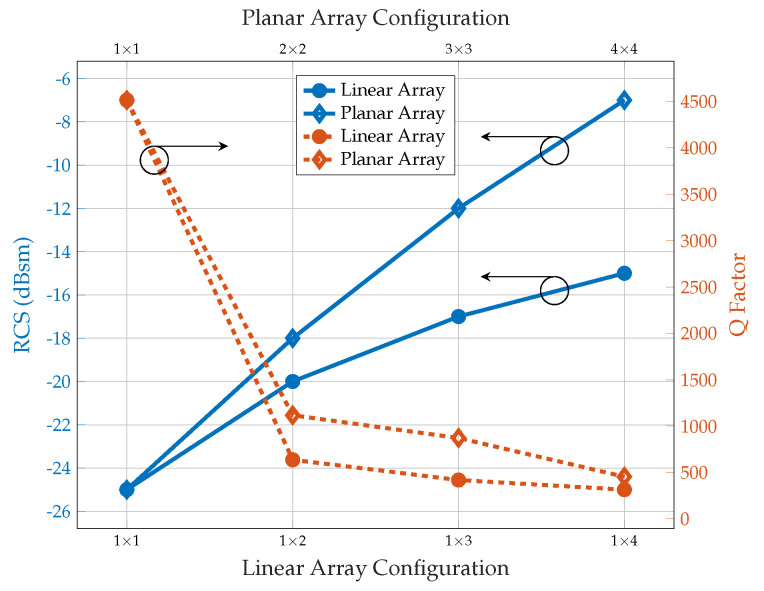
Simulated Q-factor variations of linear and planar array configurations with respect to RCS.

According to the simulated Q of the different configurations of the linear and planar array, as depicted in Figure 15, the level of RCS increases with an increase in the number of elements, but the Q-factor decreases. As the number of elements in the array increases, there is a greater possibility of electromagnetic coupling between adjacent elements despite the reduced coupling during the design phase. This coupling can result in a wider resonance and a lower Q-factor. In addition, radiation losses may become more significant. When the chipless RFID array is excited, arrays may have more radiation losses, which can contribute to a further decrease in the Q-factor.

As aforementioned, the Q-factor is generally higher in planar array configurations than in linear arrays, primarily due to stronger mutual coupling and enhanced energy confinement. In a 2×2 planar array, each element interacts with three adjacent elements, leading to improved energy storage and reduced radiation losses. As a result, this configuration achieves sharper resonance and a narrower bandwidth, making the system more frequency selective. In contrast, a 1×4 linear array exhibits weaker coupling, with each element interacting with only two adjacent elements. This limited interaction results in faster energy dissipation and a lower Q-factor.

Taking into account the same footprint area of the tag, the linear array 1×4 and the planar array 2×2, it is noticed that the linear array shows a higher RCS value of 2 dBsm but a lower Q-factor as shown in Figure 15. Indeed, a higher Q-factor influences the detectability more than the 2 dBsm RCS difference. Furthermore, according to Figure 11b and Figure 12b, the RCS level of the planar array shows a significant improvement compared to the RCS plot of the linear array. Therefore, considering the above-simulated results, planar array configurations based on a Pi-shaped resonator have been identified as the most suitable configuration for various applications and are fabricated to validate the simulation results.

Additionally, our findings strongly align with the Chu–Harrington theory [34]. It is noted that as the array size increases, a decrease in the Q-factor occurs due to the broadening of the bandwidth. This suggests that with an increase in the effective electrical size of the resonator, a shift in the distribution of stored energy takes place, leading to a reduction in resonance sharpness. The increase in bandwidth associated with a larger array is consistent with the fundamental trade-off described in antenna theory, where it is acknowledged that electrically larger structures tend to exhibit improved radiation or scattering efficiency, albeit at the cost of a lower Q-factor. This behaviour aligns with the classical bandwidth limitations observed in electrically small antennas and further supports the applicability of the Chu–Harrington limit in the context of chipless RFID arrays.

## 6. Experimental Validation and Discussion

### 6.1. RCS of Planar Array Configurations

**Figure 16:**
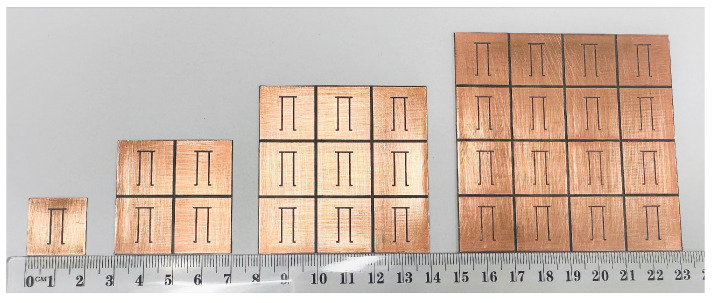
Fabricated planar array configurations of Pi-shaped resonator unit cell, 2×2 array, 3×3 array and 4×4 array.

After investigating the simulation results of the linear and planar array configurations, chipless RFID tags of the planar array configuration are fabricated to validate the simulation results because the planar arrays show better RCS improvement and detectability than the linear configurations. We fabricate four planar array configurations using a milling machine, as illustrated in Figure 16, with a plastic ruler for the actual size comparison of the tags.

**Figure 17:**
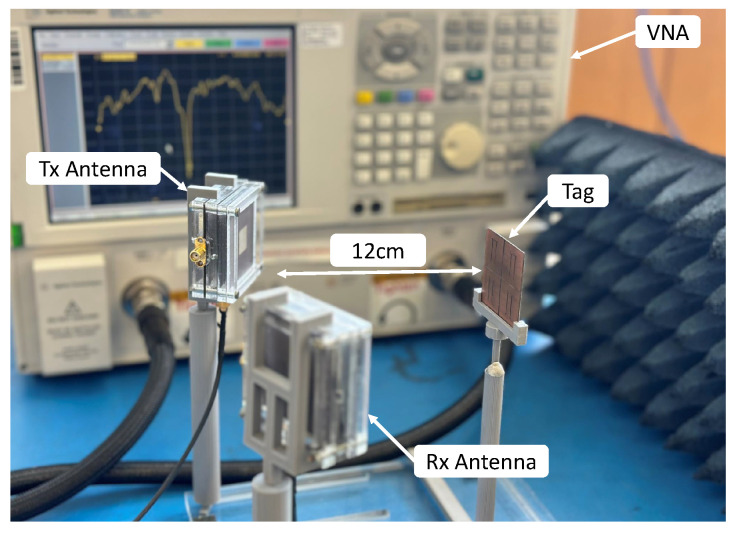
Collocated bistatic measurement setup using a VNA with dual-polarised patch antennas.

Figure 17 shows the collocated bistatic measurement setup to measure the chipless planar array tags using the Keysight E8361A vector network analyser (VNA). Vertically polarised patch antennas with 9 dBi gain are connected to the VNA using ports 1 and 2, and more technical information about the antenna can be found in [35]. We use monostatic and collocated bistatic antenna configurations in this experiment to obtain the measurements. In the monostatic configuration, only one antenna is connected to VNA using port 1. This is one of the most common measurement arrangements for copolar chipless RFID tags since it only transmits the signal in the same direction as received (i.e., the E plane is vertical in both TX and RX). The VNA output power is adjusted to 0 dBm across the whole frequency band, with an averaging function active for 20 measurements to eliminate minor variations.

**Figure 18:**
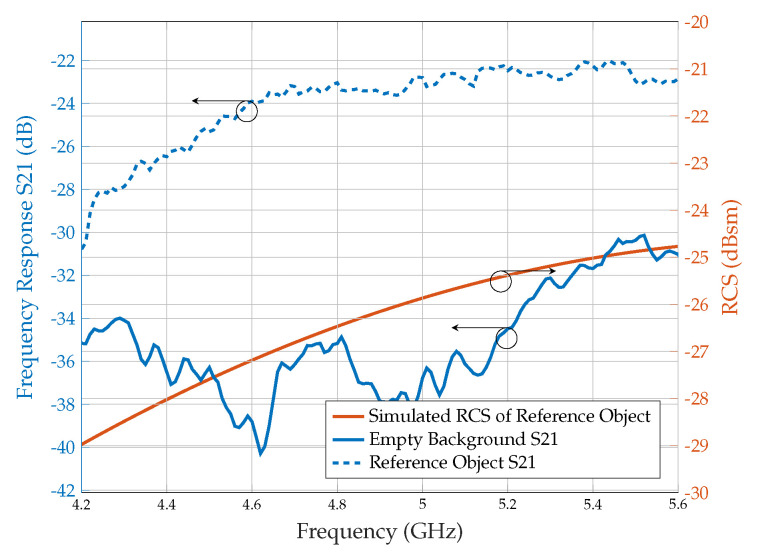
Measured frequency response of empty background, reference object using collocated bistatic configuration, and simulated RCS of a reference object.

For the RCS measurements, background subtraction is necessary to cancel out the antenna’s effect and environmental noises on the received signal. In addition, a reference object, such as a metal plate of the same size as the tag, is used to scale the subtracted signal. Therefore, as shown in Figure 18, the S21 parameter is first captured in the empty background (e.g., without the tag). Tags holding forms should be included in the empty backdrop measurement because we only require the RCS of the tag. Secondly, we measure the S21 of a reference object with a known RCS. For example, aluminium plates with the exact sizes of the array tags are used as reference objects. Finally, the RCS is calculated using (3). Furthermore, the expression for the RCS of reference object $\sigma_{\mathrm{ref}}$ is generated from the simulation of CST Microwave Studio (academic version 2022) rather than using an empirical formula to obtain accurate results as shown in Figure 18.

**Figure 19:**
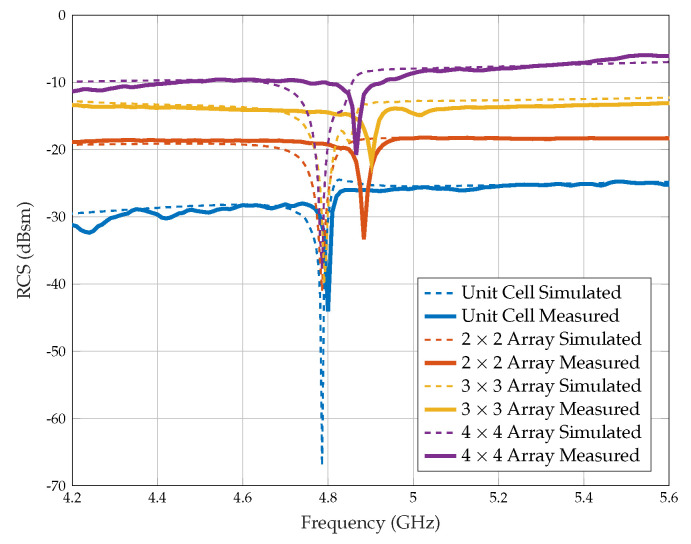
Measured and simulated RCS comparison of the planar array configurations using collocated bistatic configuration.

The results of the collocated bistatic RCS experiment are presented in Figure 19 and Table 4. We find that the measured RCS values are very close to the simulated values, so the tags can be easily detected and identified using a radar system. However, we do notice some minor differences between the resonance frequencies of the measured and simulated results, which could be due to various factors, such as the fabrication process or measurement conditions.

In this scenario, the unit cell tag has a resonance of 4.80 GHz instead of the expected resonance of 4.78 GHz. This results in a slight difference of Δf = 0.02 GHz. Additionally, the other array configurations, including 2 × 2, 3 × 3, and 4 × 4 arrays, also show deviations from the simulated values of 0.10, 0.11, and 0.08 GHz, respectively as shown in Table 4.

### 6.2. Detectability of Planar Array Configurations

#### 6.2.1. Linear Detectability

**Figure 20:**
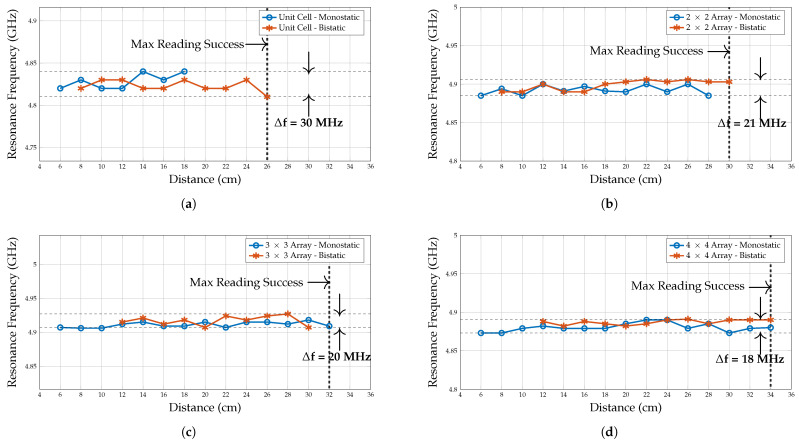
Variation in the resonance frequency of planar array configurations in monostatic and collocated bistatic antenna configurations. (**a**) Unit cell. (**b**) 2×2 array. (**c**) 3×3 array. (**d**) 4×4 array.

First, to properly analyse the linear detection performance of various array configurations, it is crucial to ensure that the resonance frequency remains stable regardless of the linear distance between the chipless RFID tag and the reader. This stability can be confirmed by examining the frequency signature of the array. For example, in Figure 20a, it is shown that the resonance frequency of the unit cell measured in the monostatic configuration remains uniform up to a distance of 18 cm from the reader. However, beyond this distance, the resonance frequency begins to deviate from the intended frequency. However, the bistatic measured resonance frequency remains stable up to 26 cm and then begings fluctuating.

In contrast, Figure 20b,c indicate that the resonance frequency of the 2 × 2 and 3 × 3 array configurations remains steady up to a distance of 30–32 cm, and variation is around 21 and 20 MHz, respectively. However, the bistatic measured resonance frequency tends to fluctuate at shorter distances, primarily because of the difficulty of focusing the antenna’s main lobe into the chipless tag. Thus, it is removed from the measured data.

Likewise, in the 4 × 4 array configuration, as shown in Figure 20d, the resonance frequency remains stable up to 34 cm and shows a lower frequency variation of 18 MHz compared to the other two arrays. Similarly, the resonance frequency fluctuates at shorter distances; hence, it is neglected. Apart from the difficulty of focusing the antenna’s main lobe into the chipless tag, the structural mode RCS is significant at a shorter distance.

**Figure 21:**
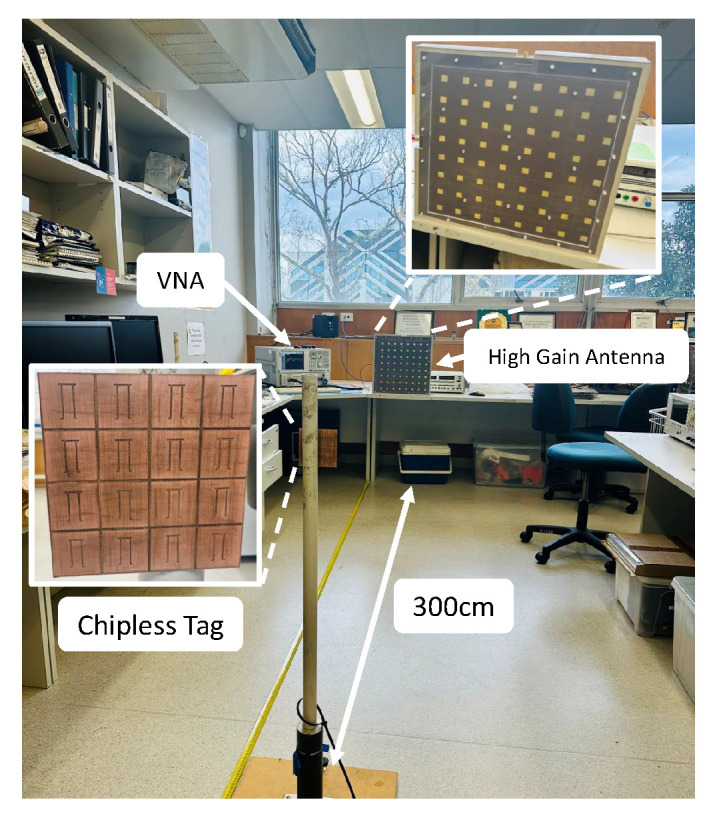
Extended reading range measurement setup using a high-gain antenna.

In order to investigate the extended linear reading range of a chipless RFID planar array, a comprehensive experimental investigation is conducted using a high-gain dual-polarised ultra-wideband array antenna [36]. This antenna has an impressive 26 dBi gain and operates in the 4.20 to 7.10 GHz frequency range. The study aims to determine the effectiveness of different array configurations in improving reading range as shown in Figure 21.

**Figure 22:**
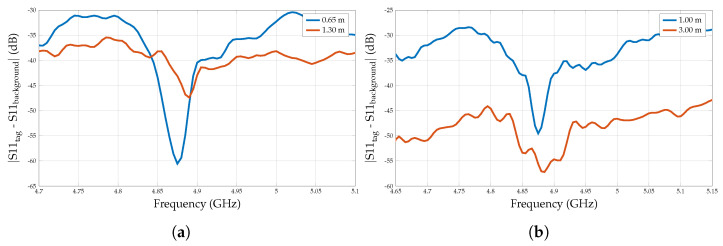
Extended reading range measurements. (**a**) 2 × 2 array. (**b**) 4 × 4 array.

According to Figure 22a, this experiment demonstrates that the resonance dip of the 2 × 2 array becomes noticeable at around 4.90 GHz when the distance is between 0.65 and 1.30 m. However, this resonance fades away when the reading distance increases to 2.00 m. On the other hand, the 4 × 4 array demonstrates a sustained resonance dip that is observable up to 3.00 m as shown in Figure 22b. This sustained resonance can be attributed to the higher RCS of the 4 × 4 array. However, as summarised in Table 5, there are minor resonance frequency variations when the distance increases. This variation is because the effect of the environmental noises becomes more prominent when the distance increases, and there is a slight variation in the test setup. This variation can be minimised when the measurements are conducted in the anechoic chamber, implementing a VNA averaging function, and manually triggering the measurements instead of continuous triggering.

#### 6.2.2. Spherical Detectability

**Figure 23:**
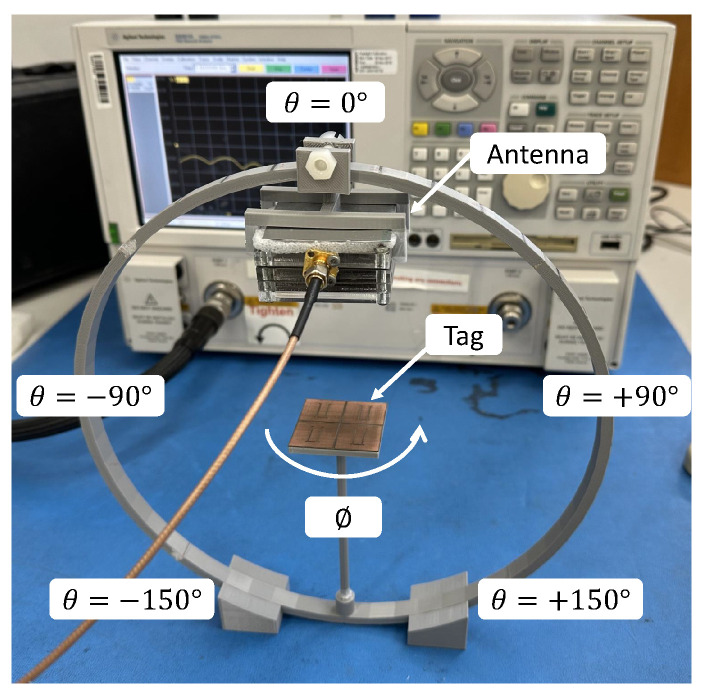
A 3D spherical identification of chipless RFID tag measurement setup.

Next, the characterisation of the spherical detection zone of the chipless RFID tags is carried out using the monostatic radar configuration as shown in Figure 23. The 3D printed antenna rotating system inspired by [37] is used to rotate the dual-polarisation patch antenna. The elevation interval, *θ*, ranges from −150° to +150° with a step size of 10° and the azimuth angle, *ϕ*, spans from 0° to +180° with a step size of 30°.

**Figure 24:**
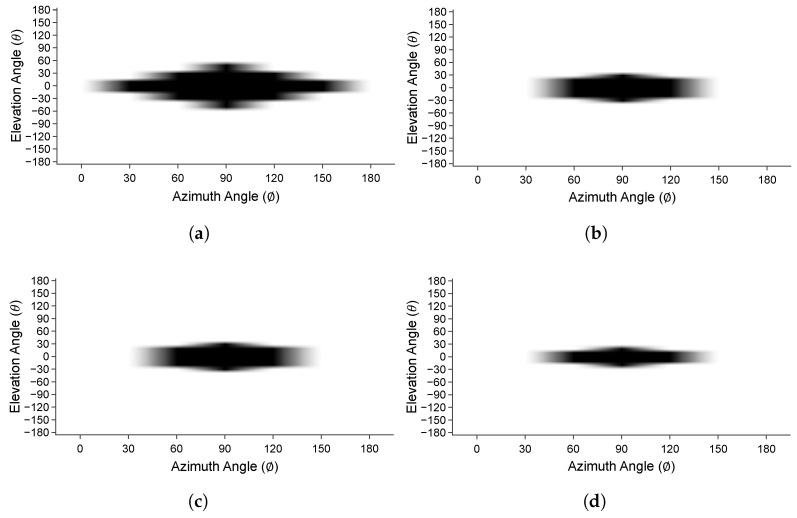
The 3D spherical detection success of chipless RFID tags. (**a**) Unit cell. (**b**) 2×2 array. (**c**) 3×3 array. (**d**) 4×4 array.

The success of chipless RFID tag identification in 3D spherical coordinates is presented in Figure 24. In the unit cell tag, as shown in Figure 24a, the successful reading range of the tag varies from −60° ≤ *θ* ≤ +60°. Reading is unsuccessful for other elevation angles due to the ground plane of the chipless RFID tag. Similarly, all other array configurations show successful readings where θ = 0°ϕ = 90°. However, the success of the 3D spherical identification narrows as the number of elements of the array increases as shown in Figure 24b–d. The far-field RCS pattern narrows and concentrates on the array’s middle (i.e., less scattering) as array elements increase, causing a narrower spherical reading range.

### 6.3. Q-Factor of Planar Array Configurations

**Figure 25:**
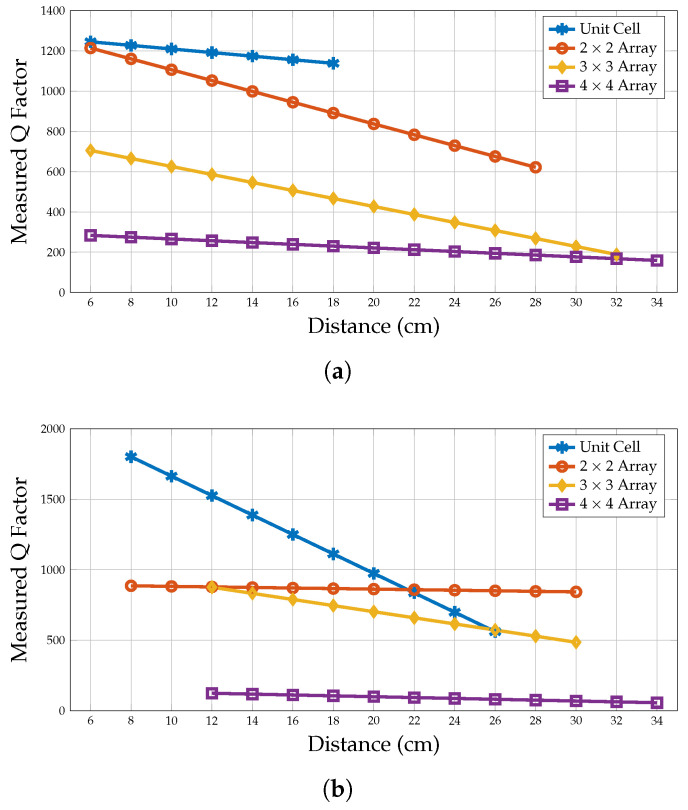
Measured Q-factor of planar array configurations. (**a**) Collocated bistatic configuration. (**b**) Monostatic configuration.

The Q-factor, a crucial parameter in planar array configurations, is measured using monostatic and collocated bistatic configurations. The linear trend line variation of the Q-factor is plotted as shown in Figure 25. However, there is a noticeable difference between the measured Q-factor and the simulated results. This difference in the Q-factor could be due to various factors, such as the quality of the measurement setup components, the alignment of the tag with the reader antenna, the reader and antenna’s efficiency, electromagnetic interference, or the properties of the material used to create the tags.

As shown in Figure 25a, the collocated bistatic measured Q-factor of unit cell tag shows the highest Q-factor, and then it is reduced when the configurations of the array increase from 2 × 2 to 4 × 4. As the number of elements in the array increases, there is a greater chance of electromagnetic coupling between adjacent elements, and it can result in a lower Q-factor. Additionally, when a plane wave interrogates the chipless RFID array, the arrays may experience more significant radiation losses, leading to a further decrease in the Q-factor. A similar behaviour can also be found in the monostatic measured Q-factors as shown in Figure 25b. Furthermore, the results of the bistatic measurements show a higher Q-factor than those of the monostatic measurements. In a monostatic setup, a single antenna is used to transmit and receive signals. While the antenna excites the tag and receives the backscattered signal, it has to handle both the transmitted and weak backscattered signals. This can result in signal degradation, reduced sensitivity, and a lower measured Q-factor due to increased losses and noise.

On the other hand, separate antennas transmit and receive signals in a collocated bistatic configuration. Each antenna can be optimised for its specific function, leading to better performance than a single antenna used in the monostatic configuration. This can result in a higher measured Q-factor due to reduced losses and improved sensitivity.

## 7. Trade-Off Analysis

The performance of chipless RFID tags based on planar array configurations is evaluated using several key parameters. These parameters include linear detectability, spherical detectability, RCS, and the Q-factor. Understanding the trade-offs among these factors is crucial to optimise the design and operation of chipless RFID systems.

### 7.1. Linear Detectability vs. Spherical Detectability

Planar array configurations can exhibit different behaviours in terms of linear and spherical detectability. Larger array configurations enhance linear detectability since they can scatter electromagnetic waves better and more efficiently in a specific direction, resulting in more stable resonance frequencies over longer distances.

In contrast, small array configurations enhance spherical detectability since they can scatter electromagnetic waves more efficiently in multiple directions. This allows for successful tag identification across a broader range of angles. However, increasing the array size also concentrates the radiation pattern, limiting the spherical detection zone at certain angles.

### 7.2. RCS vs. Q-Factor

The RCS of a chipless RFID tag determines its scattering characteristics when illuminated by incident electromagnetic waves. Therefore, larger arrays show a higher RCS, indicating a more substantial scattering effect, which improves tag detection and identification in radar systems. However, it is essential to note that larger arrays also increase structural mode RCS, which may lead to false tag identification in crowded RFID environments. On the other hand, the Q-factor reflects the quality of resonance in the chipless RFID tag. Smaller arrays, such as a unit cell or 2 × 2 array, show a higher Q-factor, which signifies a narrower resonance bandwidth and a sharper resonance peak, leading to improved frequency stability and discrimination against noise and interference. However, achieving a higher Q-factor usually requires careful design considerations and higher dielectric substrate materials, which may result in more significant manufacturing costs.

### 7.3. Optimising Trade-Offs

Optimising the trade-offs among the linear detectability, spherical detectability, RCS, and Q-factor involves balancing the conflicting requirements of each parameter to meet specific application needs. For instance, larger array configurations with better linear detectability may be preferred in critical long-range linear detection applications, even if they sacrifice spherical detectability and Q-factor.

Conversely, smaller array configurations offering enhanced spherical detectability may be more suitable in applications requiring robust performance across various angles despite potentially lower linear detectability and RCS.

**Figure 26:**
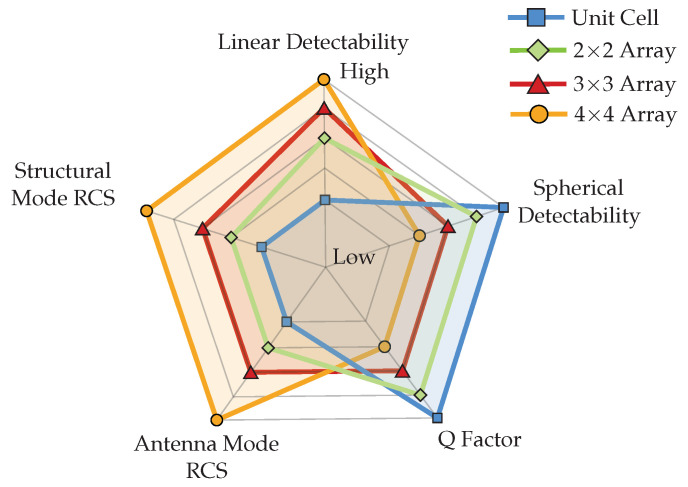
Trade-off summary of studied parameters of chipless RFID planar array configurations.

Furthermore, optimising the trade-offs may involve iterative design iterations and simulations to find the most suitable array configuration that meets the desired performance criteria within the application environment and budget constraints. Ultimately, understanding and navigating the trade-offs between linear detectability, spherical detectability, RCS, and the Q-factor is essential for the triumphant design and implementation of chipless RFID systems that are fine-tuned to meet specific application requirements as illustrated in Figure 26.

### 7.4. Future Optimised Array Design Considerations

In assessing the trade-offs of various array configurations for chipless RFID tags, it is evident that the planar array structure enhances the RCS but decreases the Q-factor compared to unit cell and linear arrays. This reduction in the Q-factor arises from the simple replication of unit cells without optimising their spatial arrangement and coupling effects. More advanced designs could mitigate these issues while retaining improved detectability.

Future designs should explore different array configurations to reduce mutual coupling and enhance resonance. Techniques such as staggered placements of resonators, varying element spacing, or utilising phase-tuned structures could improve the Q-factor while maintaining or increasing RCS. Additionally, incorporating metamaterial-inspired designs or defected ground structures (DGSs) may enhance resonance stability and frequency selectivity.

Another vital consideration is the array compactness for practical applications. The current planar design requires considerable surface area, which may not be feasible for space-constrained scenarios. Future research should focus on creating compact, high-performance arrays that retain detection and scattering efficiency. Techniques like using higher-permittivity substrates or innovative folding methods could provide a more space-efficient design. These enhancements can improve the trade-offs among linear and spherical detectability, RCS, and Q-factor, making chipless RFID systems more suitable for real-world applications.

## 8. Conclusions

Our research studied the performance and trade-offs of chipless RFID tags using linear and planar array configurations. We created a Pi-shaped resonator with a high Q-factor to design six different array configurations, including three linear arrays (1×2, 1×3, and 1×4) and three planar arrays (2×2, 3×3, and 4×4). Our simulations showed that planar arrays outperformed linear arrays in terms of the RCS and Q-factor. Through experimental validation and analysis of fabricated planar arrays, we found that the simulated and measured RCS values were in close agreement, indicating potential for reliable tag detection using radar systems. We also discovered that the trade-offs between linear detectability and spherical detectability were influenced by the array size. While larger arrays (e.g., 4×4 array) offered better linear detectability, smaller arrays (e.g., unit cell and 2×2 array) provided enhanced spherical detectability. Moreover, we highlighted the impact of the array configuration on the Q-factor, revealing the trade-offs between resonance quality and scattering efficiency. Understanding and optimising these trade-offs is crucial for tailoring chipless RFID systems to meet specific application requirements. Our study laid the foundation for developing more efficient and reliable chipless RFID systems customised to specific application needs by elucidating the trade-offs among performance parameters such as detectability, the RCS, and the Q-factor.

## Figures and Tables

**Table 2:** Summary of linear array configurations.

Resonator Configuration	RCS Level (dBsm)	Normalised RCS Level (dBsm/Cells)	Resonance Frequency (GHz)	Simulated Q-Factor
Unit cell	−25	−25.00	4.78	4515
1×2 Array	−20	−10.00	4.75	637
1×3 Array	−17	−5.77	4.75	418
1×4 Array	−15	−9.02	4.75	314

**Table 3:** Summary of planar array configurations.

Resonator Configuration	RCS Level (dBsm)	Normalised RCS Level (dBsm/Cells)	Resonance Frequency (GHz)	Simulated Q-Factor
Unit cell	−25	−25.00	4.78	4515
2×2 Array	−18	−24.02	4.78	1117
3×3 Array	−12	−21.54	4.79	872
4×4 Array	−7	−19.04	4.78	454

**Table 4:** Summary of simulated and measured results of planar array configurations.

Resonator Configuration	Simulated			Measured			ΔRCS (dBsm)	Δf (GHz)	ΔQ
	RCS Level	Resonance Freq. (GHz)	Q Factor	RCS Level	Resonance Freq. (GHz)	Q Factor			
Unit cell	−25	4.78	4515	−25	4.80	1224	0	−0.02	−3291
2×2 Array	−18	4.78	1117	−18	4.88	784	0	−0.10	−333
3×3 Array	−12	4.79	872	−13	4.90	525	+1	−0.11	−347
4×4 Array	−7	4.78	454	−8	4.86	359	+1	−0.08	−95

**Table 5:** Summary of extended reading range measurements.

Resonator Configuration	Measured		Simulated Resonance Frequency (GHz)	Δf (GHz)
	Distance (m)	Resonance Frequency (GHz)		
2×2 Array	0.65	4.88	4.78	+0.10
	1.30	4.89	4.78	+0.11
4×4 Array	1.00	4.88	4.78	+0.10
	3.00	4.89	4.78	+0.11

## Data Availability

All relevant data are within the paper.
